# Assessment of Prediagnostic Experiences of Black Women With Endometrial Cancer in the United States

**DOI:** 10.1001/jamanetworkopen.2020.4954

**Published:** 2020-05-15

**Authors:** Kemi M. Doll, Bridgette Hempstead, Julianna Alson, Liz Sage, Danielle Lavallee

**Affiliations:** 1 Department of Obstetrics and Gynecology, University of Washington School of Medicine, Seattle; 2 Cierra Sisters, Seattle, Washington; 3 Department of Surgery, University of Washington School of Medicine, Seattle

## Abstract

**Importance:**

Black women with endometrial cancer have a 90% higher mortality rate than white women with endometrial cancer. The advanced disease stage at which black women receive a diagnosis of endometrial cancer is a major factor in this disparity and is not explained by differences in health care access.

**Objective:**

To describe the prediagnostic experiences of symptoms and symptom disclosure among black women with endometrial cancer.

**Design, Setting, and Participants:**

This community-engaged qualitative study developed an interview guide to collect data during semistructured interviews among a sample of 15 black women with endometrial cancer in the United States. Interviews were conducted in person or via a secure conferencing platform. An exploratory and descriptive content analysis was performed using iterative rounds of inductive coding, case summaries, and coanalysis with community input to identify emergent themes. Data were collected from October 3, 2017, to April 15, 2019, and the descriptive content analysis was performed from October 11, 2017, to May 6, 2019.

**Main Outcomes and Measures:**

Beliefs, interpretations, and experiences of black women with endometrial cancer from symptom onset to diagnostic confirmation of cancer.

**Results:**

Participants included 15 women who self-identified as black or African American and ranged in age from 31 to 72 years. Eight participants lived in the Puget Sound region of Washington, 2 participants lived in California, and 1 participant each lived in Alabama, Michigan, Louisiana, Georgia, and New York. Twelve participants were receiving adjuvant therapy during the study, which indicated that they were either in a high-risk group and/or had advanced-stage disease. Thirteen participants had health insurance at the time of symptom onset, and all participants had elected to receive cancer treatment. Participants described knowledge gaps and silence about menopause, misinterpretation of vaginal bleeding, and responses by first-line health care practitioners that were not aligned with the risk of endometrial cancer among black women in the United States.

**Conclusions and Relevance:**

The responses of interviewed black women with endometrial cancer suggest that several mechanisms may be associated with a delay in care before diagnosis among this high-risk population and represent modifiable factors that may be useful in the development of targeted interventions to improve the rates of early diagnosis among black women with endometrial cancer.

## Key Points

**Question:**

What are the beliefs, interpretations, and experiences of black women with endometrial cancer with regard to vaginal bleeding and the disclosure of this symptom in health care settings?

**Findings:**

In this qualitative study of the interview responses of 15 black women with endometrial cancer in the United States, participants described knowledge gaps and silence about menopause, misinterpretation of vaginal bleeding, and responses from first-line health care practitioners that were not aligned with the risk of endometrial cancer among black women in the United States.

**Meaning:**

Among participants in this study, several aspects of the prediagnostic experience suggested novel modifiable factors that may be useful in the development of targeted interventions to improve the rates of early diagnosis among black women with endometrial cancer.

## Teaser

This qualitative study uses semistructured interviews and an adapted health belief model to describe the beliefs, interpretations, and experiences of symptom onset, symptom disclosure, and diagnosis among black women with endometrial cancer.

## Introduction

Racial disparity in endometrial cancer survival rates is substantial and has increased during the past decade.^1^ Endometrial cancer is the most common gynecologic cancer and is diagnosed in 1 in 37 women in the United States.^1^ Black women with endometrial cancer have a 90% higher 5-year mortality risk compared with white women, with a 5-year mortality rate of 39% among black women compared with 20% among white women.^2^ This higher mortality rate is associated in part with disparities in cancer stage at diagnosis; only 53% of black women receive an early diagnosis.^1^

Most cases of endometrial cancer are diagnosed in early stages owing to the early onset of clear symptoms, such as postmenopausal bleeding or markedly abnormal premenopausal bleeding. Although national guidelines for diagnostic examinations of postmenopausal bleeding exist,^3,4^ the guidelines assume that patients promptly report symptoms and that health care practitioners take immediate action. Delays in medical care in response to these symptoms may result in potentially untreated cancer. Yet, to date, little is known about the ways in which black women recognize, interpret, or experience postmenopausal bleeding.^5^ Although cancer stage at diagnosis has been reported as a major factor in the mortality gap between black and white women,^6^ few studies have focused on identifying modifiable factors.^7,8,9^ To address this gap, we investigated the beliefs, interpretations, and experiences of symptom onset and diagnosis among black women aged 31 to 72 years with endometrial cancer.

## Methods

This qualitative community-engaged study was performed as a partnership between an institutional research team (led by K.M.D.) and a community partner (B.H.) who is the founder and chief executive officer of Cierra Sisters, a cancer support and advocacy organization for African Americans in the Puget Sound region of Washington. The process of community engagement and the previous experience and positionality of the research team are detailed in a previous publication.^10^ In brief, we used the tools of storytelling, goal setting, and iterative collaboration to achieve 3 goals: (1) building an equitable researcher and community member (K.M.D. and B.H., respectively) partnership, (2) codeveloping the qualitative study, and (3) successfully securing research funding. This study adhered to the Consolidated Criteria for Reporting Qualitative Research (COREQ) reporting guideline.^11^ The study was reviewed by the institutional review board of the University of Washington and was deemed exempt based on the University of Washington Human Subjects Division criteria for including interactions involving only interviews with adequate provisions to protect the privacy of participants and maintain the confidentiality of the data. All participants provided verbal consent to be recorded and to have their data and responses published. Each participant received a $50 gift card for participation.

Community involvement occurred actively, with collaboration throughout the design, recruitment, data collection, and data interpretation phases. From September 6, 2017, to April 2, 2019, we recruited a purposive sample of 15 black women who had been diagnosed with endometrial cancer. A sample of 15 women was our a priori target given the likelihood of thematic saturation at this sample size^12^ and the expectation that recruitment would be resource intensive given the lack of a visible community of black women with endometrial cancer. Recruitment occurred via several mechanisms: direct contact with the gynecology-oncology clinics in the University of Washington medical system, outreach through the social networks of our community liaison and the Cierra Sisters organization, dissemination of study information on social media and on the newly formed Endometrial Cancer Action Network for African-Americans website,^13^ and posts on a general research recruitment website developed by the Institute of Translational Health Sciences at UW Medicine.^14^ Individuals were eligible for participation if they were 18 years or older, had a current or previous diagnosis of endometrial cancer (assessed through a series of confirmatory questions), self-identified as black or African American, were willing to discuss personal experiences, and had sufficient English language proficiency (assessed during initial contact).

The interview guide (Table 1) was developed with 3 organizing frameworks in mind and thus focused on the defined interval from symptoms to diagnosis,^15^ the ways in which beliefs were used to make health decisions,^16^ and the association of previous racialized experiences with actions and beliefs.^17^ The interviews focused on 3 events: menopause (to provide a knowledge base for participants’ interpretations and actions), onset of vaginal bleeding, and diagnostic experience after symptom disclosure. Questions were predominantly open ended (eg, “Tell me what menopause was like for you”), with optional probes (eg, “How did your menstrual periods change?”) included to ensure consistent and complete data collection. A few directed questions (eg, “Did your medical practitioner ever mention your race during this discussion?”) were also included. A glossary of medical terms was developed for interviewer and participant reference as needed.

**Table 1.** Summary of Interview Guide^a^

Interview guide section	Sample questions
Before diagnosis	Tell me about what menopause was like for you.
	How did you know what menopause would be like or what to expect?
	Tell me about any vaginal bleeding, spotting, or discharge you had before your diagnosis.
During examination	Tell me about what happened when you first told a medical professional.
	Tell me about what medical advice or recommendations you were given after reporting your symptoms.
	Tell me about the tests that led to your diagnosis.
After diagnosis	Tell me about the time you first received news of your diagnosis.
General reflection	What would you tell other women who may experience new vaginal bleeding after menopause?
	Looking back, is there anything you wish you knew or had been told about menopause or bleeding?

^a^ The complete interview guide with all questions, including probes and directions for interviewers, can be found in a previous publication.^10^

The interview guide was developed through several iterations within the community partnership, and both the development process and the final interview guide are available in a previous publication.^10^ The Figure illustrates our conceptual framework, which was adapted from the health belief model^16^; this model suggests that 6 factors are associated with an individual’s health behavior: risk susceptibility, risk severity, benefits to action, barriers to action, self-efficacy, and cues to action. Two authors (K.M.D. and B.H.) conducted and recorded interviews in person (n = 7) or via a secure conferencing platform (n = 8). Interviews were transcribed verbatim, verified against the recordings, and deidentified. The participants were deidentified to the research team by stripping the transcripts of any names, uploading the transcripts to the coding software by participant number, and storing the name-to-patient number linkage document in a separate file.

**Figure.**
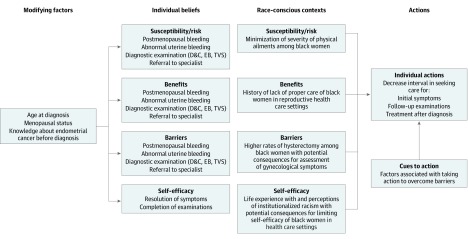
Adapted from Health Belief Model for Knowledge, Attitudes, and Beliefs Among Black Women With Endometrial Cancer For each component of individual belief, the health belief model^16^ was adapted to include relevant race-specific contexts for US black women in health care settings. For example, susceptibility to or risk of developing a symptom among black women may be associated with a race-specific context, such as the US health care environment, in which the severity of physical ailments among black women has been reported to be minimized.^18,19,20,21^ D&C indicates dilation and curettage; EB, endometrial biopsy; and TVS, transvaginal ultrasound.

We conducted an exploratory descriptive content analysis^22^ by first developing a codebook through an iterative process; using menopause, symptom onset, and diagnostic experience as a loose structure, we performed open inductive coding of the first 3 interviews, reviewing the data for ideas and organizing categories to generate the preliminary codebook. We then reviewed previous literature on the menstrual and menopausal experiences of black women^23,24,25,26,27^ and solicited feedback from our community partner (B.H.) to revise and update the codebook, which was used for all subsequent interviews.

For each transcript, a primary coder (either K.M.D., L.S., or J.A., on a rotating basis) completed the first analysis and created a case summary with relevant quotes. A secondary coder (either K.M.D., L.S., or J.A., on a rotating basis) then reviewed the transcript, the coding, and the case summary to add additional codes or points of clarification and ensure intercoder reliability. Coding was completed using Dedoose software, version 8 (SocioCultural Research Consultants). We then completed group reviews, reporting each case summary and identifying common themes until we reached saturation (ie, the point at which no new data was being obtained) at interview 12. Our community partner (B.H.) reviewed all case summaries, provided notes from the interviews themselves, and shared feedback and interpretations during group review meetings. Content areas with minimal or conflicting data were identified.

In lieu of performing individual member checking (which is not recommended to improve rigor),^12^ we incorporated participant feedback (which is important to ensure the trustworthiness of a community-engaged process)^28^ by adding targeted questions to the final 3 interviews. These questions were aligned with specific themes to clarify conflicting data and test the fidelity of our emergent themes (eTable in the Supplement). Data were collected from October 3, 2017, to April 15, 2019, and the descriptive content analysis was performed from October 11, 2017, to May 6, 2019.

## Results

Among 19 potential participants screened, 17 women were eligible; of those, 15 women enrolled in the study. Participants were aged 31 to 72 years and lived in the Puget Sound region of Washington (n = 8), California (n = 2), Alabama (n = 1), Michigan (n = 1), Louisiana (n = 1), Georgia (n = 1), and New York (n = 1). Twelve participants were receiving adjuvant therapy, indicating that they were either in a high-risk group and/or had advanced-stage disease. Thirteen participants had health insurance at the time of symptom onset, and all participants had elected to receive cancer treatment (Table 2).

**Table 2.** Characteristics of 15 Participants

Age range at interview, y	Age range at diagnosis, y	Adjuvant therapy status	Health insurance status at symptom onset	US geographic region
50s	50s	Chemotherapy and radiotherapy	Uninsured	Northwest
60s	60s	Chemotherapy and radiotherapy	Insured	Northwest
40s	40s	Chemotherapy and radiotherapy	Insured	Southeast
60s	60s	Unknown	Insured	Northwest
70s	60s	Chemotherapy and radiotherapy	Insured	Northwest
60s	50s	Chemotherapy and radiotherapy	Insured	Northwest
60s	60s	None	Insured	Northwest
60s	60s	None	Insured	Northwest
70s	70s	Chemotherapy and radiotherapy	Insured	Northwest
60s	60s	None	Insured	West
60s	50s	Chemotherapy and radiotherapy	Uninsured	South
60s	60s	Chemotherapy	Insured	Southeast
50s	40s	Chemotherapy	Insured	Northeast
30s	20s	Chemotherapy and radiotherapy	Insured	Midwest
60s	60s	Chemotherapy	Insured	West

The qualitative content analysis revealed the following themes: (1) unclear definition of normal vs abnormal menopause, (2) misinterpretation of bleeding symptoms in the context of previous personal or familial reproductive health events, (3) disclosure of vaginal bleeding cued by a waiting period or worsening symptoms, (4) vague responses from health care professionals that did not communicate risk, and (5) shock and surprise at the eventual diagnosis (Table 3).

**Table 3.** Key Themes and Subthemes From Qualitative Interviews of Black Women With Endometrial Cancer by Topic

Topic	Themes	Subthemes
Menopause experience	Unclear definition of normal vs abnormal menopause	Silence about menopausal bleeding
		Knowledge gaps because of hysterectomy in other family members
Onset of vaginal bleeding	Misinterpretation of bleeding symptoms in the context of previous personal or familial reproductive health events	Bleeding interpreted as resumption of menstrual cycles or continuation of menopause with no accompanying worry
		Bleeding interpreted as unknown occurrence with no cancer concern
		Bleeding interpreted as serious health issue with no cancer concern
Diagnostic experience after symptom disclosure	Disclosure of vaginal bleeding prompted by a waiting period or worsening symptoms	Cue to action/threshold for concern based on onset of heavier bleeding, other symptoms, or both
		Cue to action/threshold for concern based on onset of cramping, bloating, pain, or other symptoms
		Cue to action/threshold for concern based on personally defined waiting period
	Vague responses from health care professionals that did not communicate risk	No explicit discussion of cancer risk after first disclosure of bleeding symptoms
		Vague rationale for diagnostic tests and omission of purpose of tests
	Shock and surprise at eventual diagnosis	Extended duration of diagnostic interval potentially exacerbated by health insurance barriers
		No discussion of cancer risk throughout diagnostic process until point of diagnostic confirmation

### Menopause

Women reported a lack of clarity about whether symptoms were normal or abnormal during and after menopause. One woman stated, “Anything can happen in menopause” (patient 1), and another said, “I don't know that I had any real expectations what menopause would be like” (patient 3). Uncertainty in knowledge about this life transition was situated in the context of stigma and silence from peers, friends, and family regarding bleeding patterns and an absence of familial experiences with natural menopause owing to the prevalence of hysterectomy in the black community^29,30^ (Table 4).

**Table 4.** Supporting Quotations from Qualitative Interviews of Black Women With Endometrial Cancer by Subtheme

Subtheme	Supporting quotations
Silence about menopausal bleeding	Patient 4: Well, the thing is, black women don’t really talk so much about menopause. They talk more about hot flashes. They don’t talk about…you know, they’ll say, ‘Oh, I’m going through these hot flashes. I’m going through menopause.’ They don’t talk about the period thing, you know? That’s not something that they talk about.
	Patient 13: It was just 1 of those things where women would say, ‘Oh wait, I’m having a flash. Or I’m having a warming moment.’ And that would be it. Yeah. Because then they might have some beads of perspiration or whatever. And then 2 minutes later, they’d be fine and just keep on keeping on. It was never a real conversation.
	Patient 13: I think that’s the stigma with a lot of black women. You go through these things, and no one talks about it. Or they just assume that you know. And some people won’t ask questions because of embarrassment or out of ‘Oh, but I should know this, so let me just kind of figure it out or deal with it as it comes.’
	Patient 6: Even your friends don’t really want to talk about menopause. I think for a lot of women, too, it’s hard because it means you’re getting older. Something that you’ve had for a long time isn’t there anymore. I mean, at the same time, it’s nice that you don’t have to deal with it, but at the same time, you have to admit that you’re going into another phase of your life. And I think people have trouble with that.
Knowledge gaps because of hysterectomy in other family members	Patient 5: Truthfully, most of them [referring to friends] had had a hysterectomy. They wasn’t going through the hot flashes and things like that.
	Patient 9: My aunts and my mother had them [hysterectomies] before. They never talked about it. One of them told me, she said, ‘I never had it.’ And she was the one I talked to the most. And she said, ‘I never had menopause,’ and then when I talked to my mother, well, she had a hysterectomy, and she said she went through it overnight.
Bleeding interpreted as resumption of menstrual cycles or continuation of menopause with no accompanying worry	Patient 1: I thought the menopause was trying to finish because I didn’t have a lot of pain. I just didn’t. I figured if something was wrong, I would be hurting.
	Interviewer: Okay. Were you worried at all? Patient 10: Just that my period was coming back. That was it. Nothing more than that.
	Patient 8: I was just surprised. Just surprised. Not alarmed, because it wasn’t heavy at all. Very light. But it was there. And I’m like, ‘Is that blood?’ Like that. Because it was nothing compared to what I’d been used to since I was in my 20s. Nothing.
Bleeding interpreted as unknown occurrence with no cancer concern; bleeding interpreted as serious health issue with no cancer concern	Patient 9: I was thinking, ‘Oh, you’re too old to have a period, so something must be wrong with you to be bleeding.’ And that’s what my mom told me the next day when I called her. So I had to get up and change the bed and do all that stuff. Walking around, thinking about it, I was like, ‘Oh, Lord, don’t let something else be wrong with me.’
	Patient 6: One day, it was just like I had an instant period. I was in the store grocery shopping, and all of a sudden, I felt like a letdown. I looked down, and there was blood on the floor. I thought, ‘Oh, this isn’t normal. I should be done with this.’ I got out of the store as quickly as I could. I was in the meat department, no less [laughs].
Cue to action based on onset of heavier bleeding, other symptoms, or both	Patient 3: So later on, as it progressed, it’d gotten more heavier and I started receiving menstrual cramps, and that’s when I thought…they went on and I was like, ‘Wait a minute. I hadn’t had cramping in a long time.’ And it started getting worse, and that’s when I went in.
	Interviewer: And what made you decide to go to the doctor about it? Patient 8: Because I was cramping a lot, and I got tired of cramping. Interviewer: So it was the cramping that was more disturbing? Patient 8: Yeah. Interviewer: Than the bleeding? Patient 8: Mm-hmm [affirmative].
No explicit discussion of cancer risk after symptom disclosure to health care professional	Patient 4: I don’t know exactly the first time, but what I remember…I do remember telling the doctor about the spotting, and that’s when I was told that it’s normal, you know, it happens sometimes. It’s just that, after 6 months or so, then I’m spotting. So I just wanted to know, since I have fibroids, why am I still spotting when I’m supposed to be through menopause? And she said, ‘Well, sometimes that happens, you know?’
	Patient 4: I thought that the fibroids were the reason why [I was bleeding] since [inaudible] I’m being told that it’s normal. ‘Okay, your pap smear has come back. Your pap smear is fine. Everything’s fine. Your ultrasound was fine. Your fibroids haven’t grown.’ And that was it. So, I didn’t have any thoughts on having cancer. Then I was telling her, ‘I do have a little bit of spotting every 3 or 4 or 6 months, or something like that.’ She said, ‘Well, let’s do the pap smear. Let’s do the other testing for venereal diseases and stuff.’ And, of course, I told her about my fibroids. She said, ‘Well, that could be the cause of it. Let’s just do’ not the ultrasound…Oh, God…‘just do the testing, and then we’ll see.’ So everything came back normal, with nothing wrong.
No discussion of cancer risk throughout diagnostic process until point of diagnostic confirmation	Patient 12: Cancer never came to my mind, never entered my spirit.
	Patient 2: And when I was diagnosed, it was like the scariest thing in the world when I received that call. Let’s see, ‘Hey you tested positive for…’ It was devastating. I was truly devastated, you know?

Although many women noted that friends and family were their sources of knowledge, these statements were frequently accompanied by the judgment that such information was inadequate. Women directly communicated the inadequacy of their knowledge by expressing the desire to have known more about menopause. One participant said, “And we never…it was not enough education in the community to alert women, especially African American women, of what to expect. And what some of the changes would be. So I was very uneducational [sic] when it come to that” (patient 2). In addition, women described silence about bleeding symptoms during and after menopause that occurred in conversations throughout their lives, which also featured elements of stigma and shame. One woman relegated discussion of bleeding as inappropriate, stating, “It wasn’t a normal topic of conversation” (patient 10), while another associated silence with the larger experience of stigma and shame among black women (Table 4). Another participant elaborated, stating, **“**I’ve never really talked to a lot of people about menopause. Most people I know don’t want to discuss it. It’s as simple as that” (patient 6).

Another factor associated with the lack of knowledge regarding normal vs abnormal symptoms was the prevalence of hysterectomy in participants’ social networks, which was reported by 2 participants. Mothers, sisters, and friends were all perceived as insufficient sources of knowledge after they had undergone this procedure (Table 4).

### Vaginal Bleeding

All participants experienced abnormal or postmenopausal bleeding before receiving a diagnosis of endometrial cancer. Underlying their disparate experiences was an interpretation of bleeding symptoms in the context of their own previous reproductive health events (Table 4).

Participants expressed 1 of 3 initial interpretations of vaginal bleeding: (1) it was a sign of resumed menstrual cycles or continued menopause; (2) it represented an unknown event that was not associated with cancer; and (3) it indicated a serious health issue, including cancer. The first interpretation was the most common. Women directly compared the onset of new bleeding with their previous experiences of menopause or menstruation. One participant described thinking, “Hmm, I’m just not done with this yet” (patient 2) when referring to new vaginal bleeding that occurred several years after menopause. Another participant, also several years past menopause, interpreted her vaginal bleeding as a menstrual period, stating, “Every time my period would go away, it would come back” (patient 8). Along with this interpretation came a resulting lack of concern.

Women who expressed initial nonspecific worry understood that their symptoms were not normal, but they lacked direction regarding the potential association of these symptoms with cancer or other serious illnesses. One woman stated, “It was something wrong, but I didn’t know what.” Previous reproductive health experiences were important factors. As another woman stated, “'I had a very, very light pinkish bleeding, and that's what started me to think that something was off because I had already gone through the menopause, and I had totally stopped any periods at all” (patient 2). Another woman said that her previous experiences with heavy menstrual periods mitigated her worry (Table 4).

When women expressed an immediate interpretation of bleeding as a potentially serious health issue, they did so with reference to their previous reproductive health experiences. The increased severity of initial symptoms in contrast with previous menstrual experiences met the threshold of alarm. One woman woke up with a soaked mattress from new-onset bleeding. Another described a public experience of having an instant menstrual period (Table 4). Although few women experienced acute vaginal bleeding, our findings were supported by another participant who did not experience severe bleeding but remarked that increased bleeding severity would have met her personal threshold for taking action, saying, “And had it been heavier, I might've would've called the doctor” (patient 11).

Not all study participants experienced a natural onset of menopause before receiving a diagnosis of endometrial cancer. Among the 3 participants who had not yet experienced menopausal symptoms, 2 women noted marked changes in their menstrual patterns (eg, spotting between cycles, more severe bleeding during cycles, or both), and 1 woman, who did not note specific changes, had a history of irregular and severely heavy menstrual cycles that required the receipt of blood transfusions, which were normalized by health care practitioners, beginning at age 19 years. In response to her bleeding symptoms, she stated that she “got to the point where I quit talking about it. I just felt that, that was normal for my body…and that this is what was gonna go on. You know, I was kinda scared, but I still thought, well, none of the doctors are saying that it's anything, so I just need to, to deal with it” (patient 14).

### Diagnostic Experience

We defined a diagnostic experience as the interval from the first disclosure of symptoms to a health care practitioner to the diagnostic confirmation of endometrial cancer. This interval was characterized by 3 events: a waiting period or worsening symptoms that prompted symptom disclosure, a mismatch between the participant’s risk of cancer and the practitioner’s response, and an experience of shock or surprise at diagnosis.

#### Symptom Disclosure

We found that a common strategy among women was to endure the symptoms before reaching a personal threshold, at which point they decided that taking action was the next appropriate step (ie, the cue to action).^16^ One participant described her endurance of symptoms by stating, “Well this has got to be over soon, one day” (patient 1), while another remarked that she could “just live with it” (patient 4). Endurance of symptoms emerged across the cohort as a lack of reported action beyond the maintenance of hygiene (eg, purchasing sanitary pads).

The cues to action for symptom disclosure included a personally defined waiting period, the onset of heavier vaginal bleeding or other symptoms, or both. Regarding the waiting period, 1 individual decided to disclose her bleeding symptoms “because it had been happening for just about a year” (patient 13). Many women said that their concern increased as bleeding became progressively heavier (Table 4). Other symptoms that prompted disclosure included cramping, bloating, fatigue, and nausea, all potential signs of cancer progression. One woman noted her concern about fatigue after bleeding for more than 6 months, stating, “The fatigue was just totally not me. I mean, it was like somebody drugged me. Totally uncharacteristic of my normal self” (patient 11). Another participant described the cramping symptoms as more concerning than the bleeding (Table 4).

#### Practitioner Response

We found a pattern of health care practitioner responses after symptom disclosure that lacked explicit mention of cancer as a potential explanation for vaginal bleeding despite the fact that women reported symptoms of concern. This mismatch was noted during the women’s recounting of reassuring remarks from their practitioners (Table 4). One participant could not recall when she reported symptoms, as doing so had prompted normalization of the symptoms by her practitioner. She recalled the practitioner saying, “Well, sometimes that happens, you know?” (patient 4).

All but 1 of the women received recommendations for further diagnostic testing in the form of a transvaginal ultrasound, endometrial biopsy, or dilation and curettage procedure. The theme of practitioner response mismatch with women’s health risks was also observed in the vague rationale given for the need for diagnostic tests. Women received vague explanations, such as, “I just wanna make sure there's nothing going on in there” (patient 1). After ultrasound results indicated enlarged measurements of the endometrium (as reported by participants) that suggested cancer risk, the vagueness in descriptions continued, with one practitioner saying, “Oh, it looks like there's heavy lining in your uterine walls, so I'm just gonna send you to this doctor and have it checked out” (patient 1). No women reported receiving information that a transvaginal ultrasound was a screening and not a confirmatory test.

#### Diagnosis

The combination of reassurance and the lack of explicit discussion of cancer risk led to shock and surprise when the endometrial cancer diagnosis was received. One woman recalled the following discussion with her practitioner: “I said to her, ‘What do you think this is?’ And she said to me, ‘If I was a betting woman, we say you have cancer.’ And I was in the middle of [censored] street on a sunny day carrying my lunch that I had just picked up. And I dropped it” (patient 13).

Participants also expressed surprise that cancer could have been associated with their symptoms at all. As 1 woman expressed, “Cancer never came to my mind, never entered my spirit” (patient 12).

## Discussion

To our knowledge, this is the first study to explore the prediagnostic experiences of black women with endometrial cancer. The women in the study described knowledge gaps and silence about menopause, misinterpretation of vaginal bleeding in the context of these knowledge gaps, and responses by first-line health care practitioners that were not aligned with the high-risk status of the women. In the following paragraphs, we place these findings in the larger context of US black women’s gynecologic health and consider the ways in which, within these experiences, multiple novel factors associated with care delays may exist both before and immediately after symptom disclosure to health care professionals.

Both the nature and interpretation of symptoms are associated with the length of time from symptom onset to the perception of individual risk. The interpretation of symptoms as not new, not bothersome, or not painful has been associated with care delay.^15^ These interpretations, however, are subjective and dependent on the individual’s previous experiences and norms. The women in our study did not have a clear expectation or standard definition of menopause or an awareness of their menopause knowledge gaps, which they associated with silence regarding vaginal bleeding among family and friends and the occurrence of hysterectomy among other family members. We hypothesize that the commonality of hysterectomy among US black women (who are 3-4 times more likely to have a premenopausal hysterectomy compared with US white women)^29,30^ may be associated with a lack of familial and community communication about menopausal transition. Unclear definitions of menopause and expectations about this transition may leave black women vulnerable to labeling any experience—bleeding, pain, bloating, or fatigue—as normal.

In addition, US black women have a high prevalence of fibroids or leiomyomas,^31,32,33^ which are benign tumors of the uterus. The presence of fibroids has been associated with abnormally heavy and irregular menstrual cycles.^34^ In a previous mixed-methods study, reproductive-aged black women were reported to have a concerning level of normalization of severe bleeding symptoms.^35,36^ We hypothesize that a phenomenon may exist in which postmenopausal black women do not regard the onset of vaginal bleeding as new because they have a history of irregular menstrual cycles or as bothersome because the new bleeding and pain are less severe than they were during previous experiences in their youth. Given that pain among black people in US health care settings has been reported to be discounted and undertreated,^18,19,20,21^ it is worth considering the possibility that black women may be discounting their own pain in adaptation to this environment.

The women’s reported cues to action (ie, increased bleeding severity and pain) to seek health care did not appear to prompt an appropriate level of concern from health care practitioners given the risk of endometrial cancer among black women.^3,37^ This misalignment between health risk and practitioner response was observed in the lack of communication about cancer risk after the women disclosed their bleeding symptoms. Although it is estimated that only 10% of women with these symptoms will be diagnosed with endometrial cancer,^37^ in the context of the knowledge gaps and misdirected coping strategies we observed among the black women in our study, such minimization of cancer risk may have exacerbated the delay in diagnosis. Without a clear discussion of cancer risk, women may continue to have assumptions of normality and use coping strategies, such as endurance of symptoms, that may be associated with subsequent delays in scheduling follow-up tests or with not receiving follow-up tests at all.

### Strengths and Limitations

This study’s strengths include a geographically diverse cohort, a sample that was large enough to reach thematic saturation,^12^ and a community-engaged design, which allowed for richness, depth, and intimacy in interviews about topics that are often regarded as taboo among black women.^38^ This study also has limitations. Because this study was qualitative, the findings were not meant to be generalizable but instead aimed to clearly communicate the experiences of the participants. Our sampling strategy may have overrepresented women with health insurance and health care access and likely underrepresented the full breadth of barriers associated with household income and insurance.^39^ As a community liaison–research group partnership, we purposively did not collect extensive demographic information (eg, income strata and educational level) owing to feedback from our community partner (B.H.), who suggested that these types of questions may deter women from participating in the study and create a barrier to the establishment of a comfortable and trusting environment for the women. By identifying and including women who had already received a diagnosis of endometrial cancer, we risked recall bias. Subsequent research to translate these findings into a large-scale national survey will add quantitative context and provide information about other environmental associations with the themes reported in this study.

## Conclusions

Black women with endometrial cancer described multiple experiences that were associated with care delay independent of health care access. Given the importance of cancer stage at diagnosis to overall cancer prognosis and the lack of interventions to address higher mortality rates among black women with endometrial cancer, these findings represent an important first step toward the development of evidenced-based interventions.

## Notes

Supplement. **eTable. **Questions Added to Test Fidelity of Themes in Final Interviews
