# Supplementary material for: Assessment of Prediagnostic Experiences of Black Women With Endometrial Cancer in the United States
Source: JAMA Netw Open. 2020 May 15;3(5):e204954. doi: 10.1001/jamanetworkopen.2020.4954 (PMC7229523; doi:10.1001/jamanetworkopen.2020.4954)
Supplement: Supplement. — eTable. Questions Added to Test Fidelity of Themes in Final Interviews [file jamanetwopen-3-e204954-s001.pdf]

Supplementary Online Content

Doll KM, Hempstead B, Alson J, Sage L, Lavallee D. Assessment of prediagnostic experiences of black women with endometrial cancer in the United States. *JAMA Netw Open*. 2020;3(5):e204954. doi:10.1001/jamanetworkopen.2020.4954

eTable. Questions Added to Test Fidelity of Themes in Final Interviews

This supplementary material has been provided by the authors to give readers additional information about their work.

© 2020 Doll KM et al. *JAMA Network Open*

eTable. Questions Added to Test Fidelity of Themes in Final Interviews

**Identified themes from analysis**	**Additional interview question**
Silencing around menopause symptoms among Black women.	**Q3.** We've heard from some women that menopause, especially the symptoms, are not something that are talking about much among Black women. Was this your experience, or do you feel differently?
Interpretation of bleeding symptoms falls into one of three patterns: interpretation of bleeding as a sign of resumption of menstrual cycles or continuation of menopause without accompanied worry; bleeding representing an unknown entity that was not cancer-related; and bleeding representing something serious, including cancer.	**Q5.** When we ask women about the bleeding they had before diagnosis, they usually say one of three things: 1) They thought the bleeding was another cycle or extension of menopause; 2) They didn't know what it was, but they didn't think it was serious; 3) They didn't know what it was, but they were very worried about cancer or something serious. Do you feel any of these describe your initial feeling? **If Yes** – tell me more. **If No** – what did you think about your bleeding?
Interpretation of bleeding symptoms as related to stigmatized sexual behaviors that the participant may or may not have participated in.	**Q6.** Another thing we've heard, is that some women have heard or been told that the bleeding is related to a history of sexual partners. Did you have any thoughts or feelings like that? **If yes** – ask why.
Participants relied on "endurance" or "waiting it out" rather than "turning outward" to friends or family for support.	**Q7.** Finally, with regard to the bleeding, some women have told us they reached out to family/friends about it and some didn't. Did you talk to any family/friends about this bleeding – why or why not?
Symptom disclosure prompted by a waiting period or worsening symptoms.	**Q9.** In talking with many women, we've heard that the reason they tell their doctors about the bleeding is that it gets heavier or they start to get other symptoms like pain. Was this the case for you or not? **PROBE:** Why did you decide to report your bleeding or other symptoms at the time that you did?

© 2020 Doll KM et al. *JAMA Network Open*
